# Surgical treatment of giant plexiform neurofibroma associated with pectus excavatum

**DOI:** 10.1186/1749-8090-6-119

**Published:** 2011-09-28

**Authors:** Yi Ji, Bing Xu, Xuejun Wang, Wenying Liu, Siyuan Chen

**Affiliations:** 1 Department of Pediatric Surgery & Center of Children Medicine, Sichuan Academy of Medical Sciences/Sichuan Provincial People's Hospital, Chengdu, China; 2 Research Institute of Pediatrics, Children's hospital of Fudan University, Shanghai, 201102, China

**Keywords:** Plexiform neurofibromas, Pectus excavaum, Nuss procedure

## Abstract

Plexiform neurofibromas are benign tumors originating from subcutaneous or visceral peripheral nerves, which are usually associated with neurofibromatosis type 1. They are almost always congenital lesions and often cause the surrounding soft tissue and bone to grow aberrantly. We treated a 12-year-old boy who presented with asymmetric pectus excavaum and an anterior chest wall plexiform neurofibroma. The pectus excavaum was corrected by modified Nuss procedure, followed by simultaneous resection of the giant mass. The patient is doing well at the 4 years follow-up visit.

## 1. Background

Plexiform neurofibromas (PNFs) are benign nerve tumor resulting from aberrant growth of the cells of nerve sheath. They are usually congenital, but they may instead present during the first year as a subtle soft-tissue enlargement or a large patch of cutaneous hyperpigmentation. PNFs are generally painless, slowly growing neoplasmas. Although most neoplasms are asymptomatic, they can be particularly debilitating due to their potential to grow to very large sizes. Presenting symptoms depend on the location of the tumors. Tumors of head, neck, and face are most common, followed by facial disfigurement and lesions of the spine, extremities, and abdomen [1]. Early childhood, puberty, and childbearing age are considered to be the periods of greatest risk for disease progression. Furthermore, PNFs have a potential for transformation into highly malignant peripheral nerve sheath tumors, which occur in approximately 5% of patients [2]. Unfortunately, there is no accepted effective medical treatment for PNFs. Current management of this disease is limited to surgical resection, but they are usually difficult to completely remove and tend to regrow. Decisions about surgical treatment and frequency of follow-up must be made judiciously and individualized for each patient [3]. Previous studies found that PNFs can be associated with pectus excavatum (PE) [4,5], which is the most common chest wall malformation and one of the most frequent major congenital anomalies. Here, we present a case of giant chest wall PNF associated with PE.

## 2. Case Report

**Figure 1:**
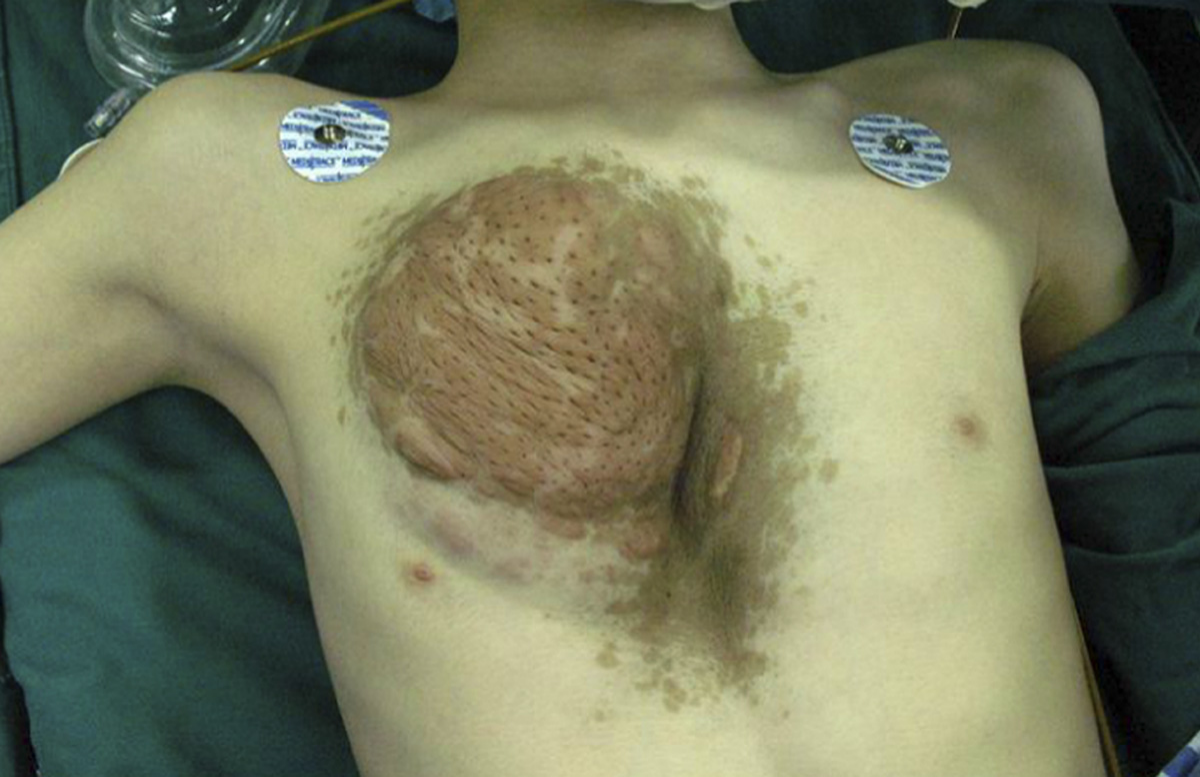
**Preoperative photograph of the chest wall**.

**Figure 2:**
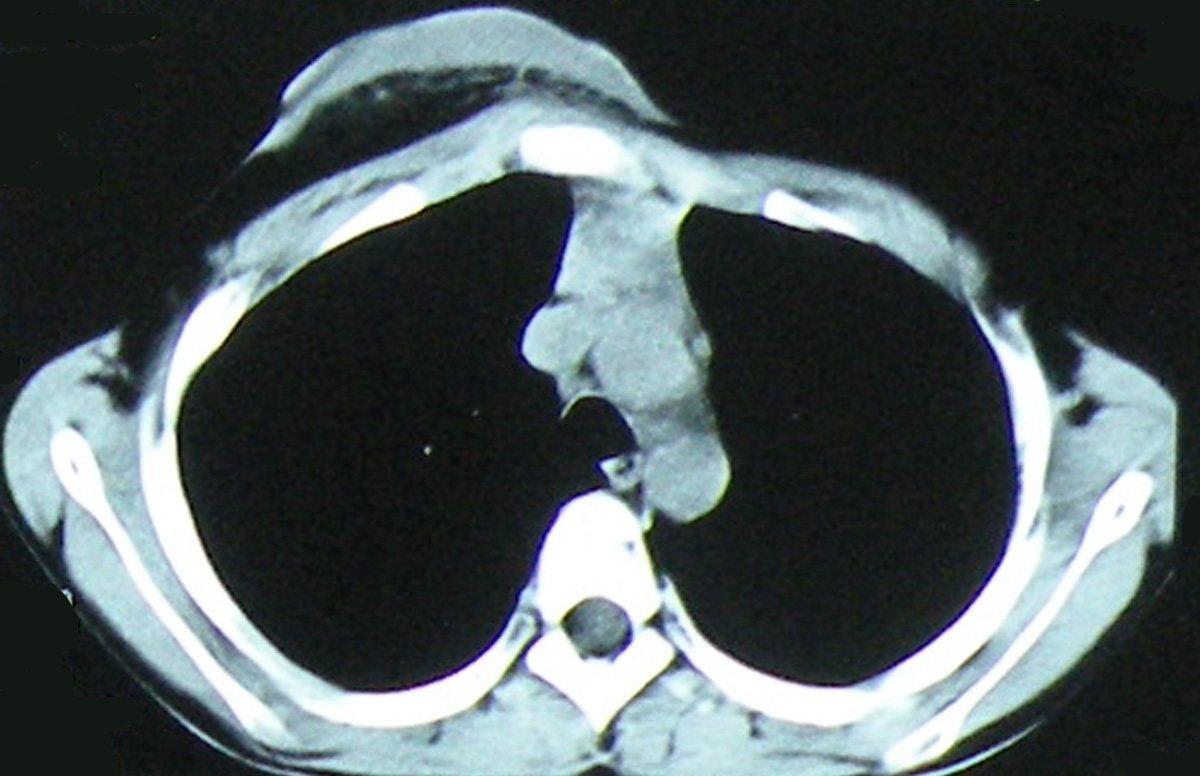
**At the level of third thoracic vertebra, CT scan shows a well-define mass in the anterior chest wall without clear signs of infiltration of surrounding structures**.

**Figure 3:**
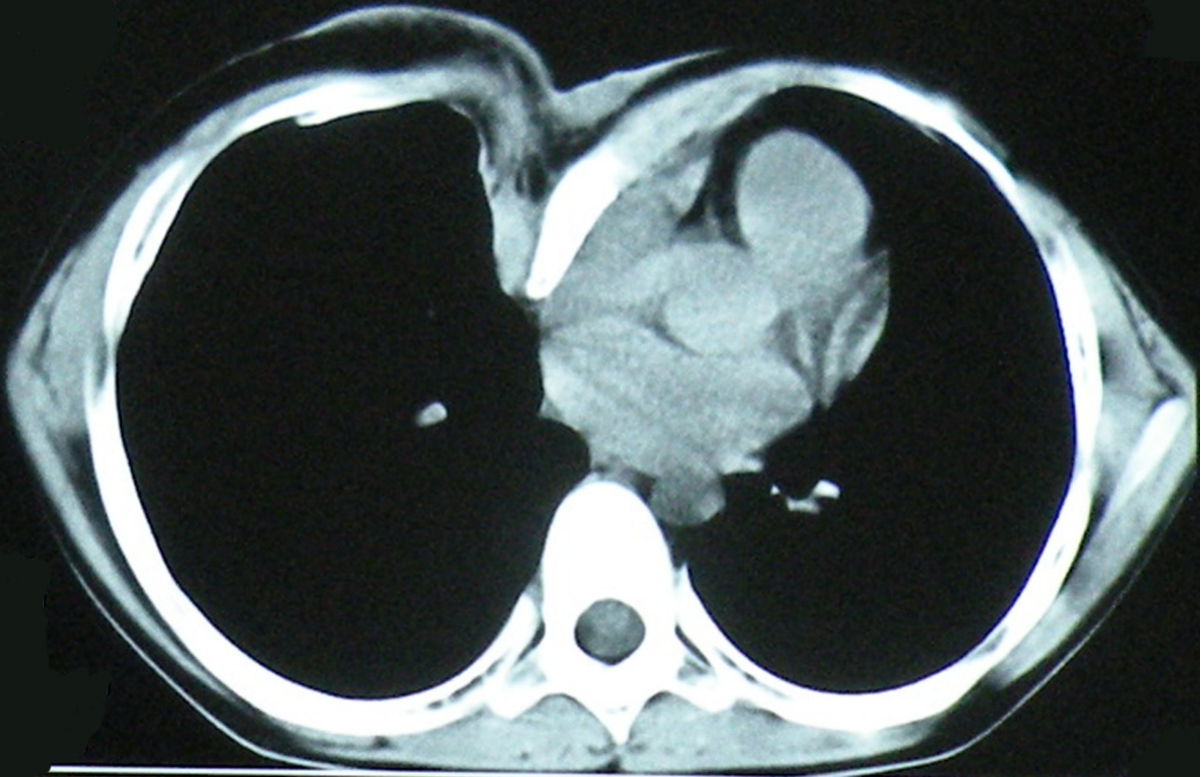
**At the level of sixth thoracic vertebra, CT scan shows marked sternal rotation (the sternum is rotated by almost 90 degrees) and depression, with a pectus index of 3.9**.

A 12-years-old boy with known neurofibromas type 1 (NF1) came to the hospital, stating that a lifelong mass on the anterior chest wall had grown steadily to its present size. He also presented with an 8 years history of increasing depression of the anterior chest wall. Systematic questioning of the patient and his parents elicited no description of significant symptoms. However, the mass was increasingly prominent and the chest wall depression became progressively worse with age, causing the patient considerable emotional distress. Physical examination revealed a soft, fixed, painless mass originating from the anterior chest wall. The mass was associated with thinning of the skin, hyperpigmentation, and it surrounded by numerous café-au-lait spots. Below the mass, a substantial chest wall depression was found (Figure 1). Pathological examination performed at another Triple A hospital 6 months ago showed a benign PNF. Computer tomographic scan showed a well-defined heterogeneous soft tissue density tumor without signs of erosion of the rib and sternum (Figure 2). The posterior depression of the chest wall was associated with severe rotation of the sternum, leading to displacement of the heart to the left (Figure 3). The findings were consistent with PNFs and an asymmetric PE.

**Figure 4:**
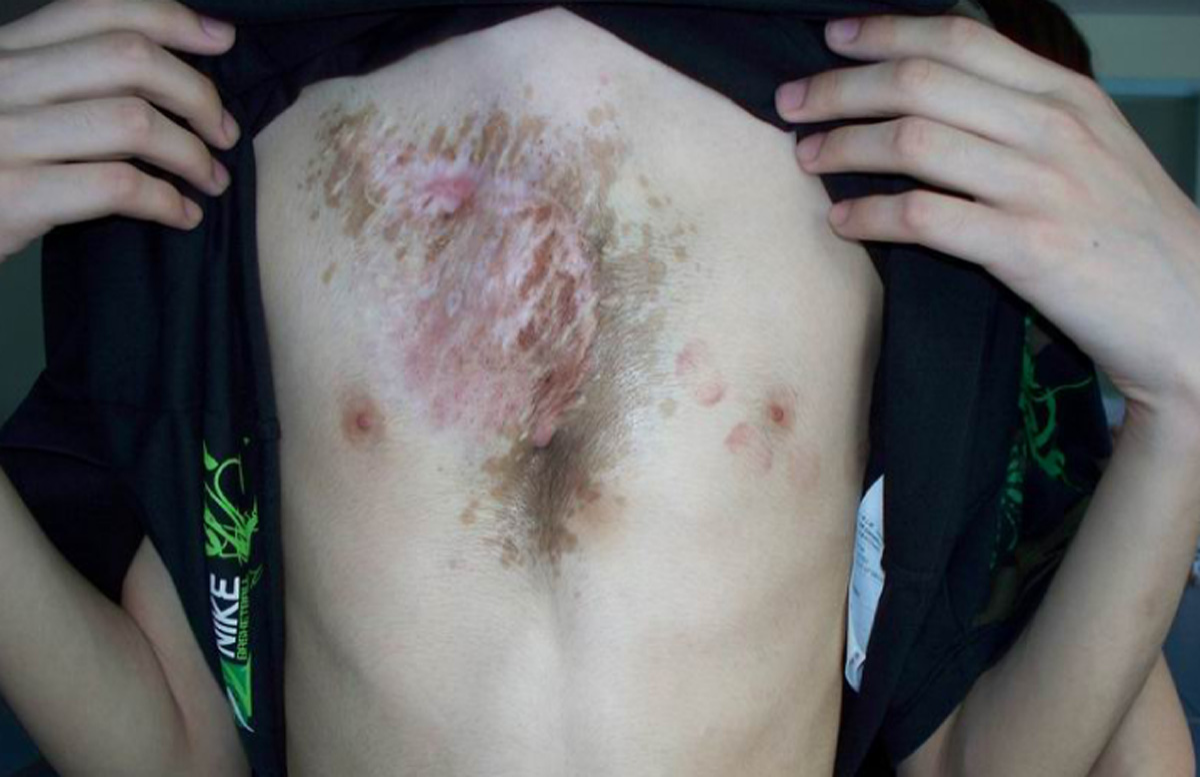
**The 4-years postoperative photograph of the chest wall**.

Given the size and location of the mass and the risk of malignant transformation, the patient was referred to plastic surgery for excision. The patient was placed in the supine position after general anesthesia. Frozen section was performed routinely at the beginning of the procedure to exclude malignancy. Surgical technique for repair of the PE was based on the modified Nuss procedure. One small vertical skin incision was made in the midaxillary line each side. A bilateral submuscular tunnel was created using a blunt dissection through bilateral thoracic skin incisions. The use of thoracoscopic visualization was not used. An appropriate introducer (Lorenz surgical Inc, Jacksonville, FL, USA) was placed into the tunnel from left side. The tip of the introducer was kept in contacting with the anterior thoracic wall while the introducer was slowly advanced across the retrosternal space. Subsequently, the introducer was slowly advanced across the opposite intercostal space and brought out through the incision on the right side. The bar was attached to the tip of the introducer and the introducer was slowly withdrawn from the tunnel followed by the bar with the bar's convexity facing posteriorly until it emerges on the contralateral side. The bar was placed directly on the ribs in the submuscular position. Both the bar and the stabilizer were fixed together onto the underlying rib with multiple interrupted non-absorbable sutures. An excision of the entire soft tissue mass was carried out right after the Nuss procedure. The visible tumor was of grayish color and of a gelatine-like consistency, stating immediately below a layer of subepidermal fat and did not extend deep to the pectoralis major. A 21.0 × 17.5 × 5.0 cm mass was successfully resected. Signs of erosion of the rib and sternum were no found. After excision of the bulky skin, two Redon drain was inserted and the wound was closed without tension. The patient received an epidural catheter for perioperative patient-controlled analgesia (PCA) for 4 days, and had a good postoperative recovery without complications. He and his parents expressed satisfaction with the cosmetic relief which achieved by the resection and reconstruction. 4 years later (1 year after the Nuss bar removal) the patient is well and free from tumor and PE recurrence (Figure 4).

## 3. Discussion

NF1 is one of the most common human genetic diseases. It has an incidence of 1 in 3000-4000 individuals and affects male and female subjects equally in all races. PNFs are considered pathognomonic for neurofibromatosis [6]. Clinically, the growth patterns of PNFs can be classified into 3 categories: superficial, displacing, and invasive. Superficial PNF arise from subcutaneous or cutaneous nerves and may remain within the upper skin layers. They occur as a result of proliferation of all supporting elements of the nerve fibers, including Schwann cell, perineurial cells, fibroblasts, blood vessels, and infiltration of mast cells. Invasive lesions infiltrate multiple tissue planes and cannot be resected without functional disturbance [7]. Malignant peripheral nerve sheath tumors sometimes arise from preexisting PNFs. Therefore, rapid growth of a PNF and onset of clinical neurologic deficits should prompt an immediate evaluation for malignant transition [3]. However, clinical alone do not allow differentiation of malignant tumors and benign PNF. Such tumors should be further examined with CT scan and/or open biopsy, and closely monitored clinical and with MRI.

Clinical management for the PNF requires a multidisciplinary approach and the use of a multi-speciality Neurofibromatosis clinic is desirable. However, current treatment options for PNF are limited to surgical intervention, and there is debate in the literature regarding the timing of operation and extent of resection. Some authors suggest that early resection of smaller tumors may minimize the extent of local involvement [1]. Others have countered that complete resection is often impossible and so cannot justify resection in asymptomatic children for whom there is a significant potential for regrowth of the residual tumor [3]. Needle et al [8] have found that age less than 10 years at operation is a prognostic for progression of the lesions. But there is no denying the fact that the resection of a giant benign tumor can be important in minimizing cosmetics deformities. The clinical presentations of PNF in an individual, in addition to the emotional burden of carrying the disease and social stigma, have a significant impact on the patient's quality of life. The PNF presented in this report had been slowly growing for 12 years, but the boy kept it hidden from public view for social reason by constantly wearing heavy clothes. The resection of the large tumor can improve patients' condition and provide good quality of life [9].

Asymmetry of the thorax (pectus excavatum, etc), kyphoscoliosis, and segmental bone hypertrophy of the leg are the skeletal abnormalities previous reported with PNFs [1,4,5]. Whether this association of pectus excavatum is a manifestation of the giant tumor or an incidental finding is unclear based on our case. Further case reports or studies are needed to establish the significant of this phenomenon.

Historically, PE was repaired with chondrosternal resection, or some combination of cartilage incision and osteotomy with or without external or internal fixation [10,11]. Minimally invasive repair of PE (MIRPE), also known as Nuss procedure, has changed the perception and understanding about surgical treatment. This method of repair offers a less traumatic procedure preservation of the costal cartilages. To prevent the occurrence of bar dislocation and cardiac perforation, submuscular bar, multiple pericostal bar fixation and bilateral thoracoscopy were advocated to use in PE patients, especially in patients with extremely deep depressions [12,13]. In this patient, the passage of introducer below the sternum was performed from the left to the right side. We maintained the tip of the introducer in direct contact with the anterior thoracic wall during passage to the other side, therefore moving from the heart and lung [14,15].

In the literature, surgical management of cardiac anomalies or other intrathoracic diseases and associated chest deformity has been well documented [16,17]. Prior thoracic surgery is not a limiting factor for the Nuss procedure [17]. In this case, we started with Nuss procedure and followed by excision of PNF. The wound was closed without tension and the pain after Nuss procedure was managed with PCA, both of which would significantly reduce the risk of wound dehiscence.

## Declaration

Written informed consent was obtained from the patient for publication this case report and accompanying images. A copy of the written consent is available for review by the Editor-in Chief of this journal.

## Competing interests

The authors declare that they have no competing interests.

## Authors' contributions

YJ was involved in the preparation of draft and finalization of the manuscript. BX advised regarding preparation of the manuscript. WYL was the chief surgeon and responsible for finalisation of the manuscript. XJW and SYC helped to draft the manuscript. All authors read and approved the final manuscript. The authors are indebted to all reviewers for their kindly reviewing of the manuscript.

## References

[B1] Nguyen R Kluwe L Fuensterer C Kentsch M Friedrich RE Mautner VF Plexiform Neurofibromas in Children with Neurofibromatosis Type 1: Frequency and Associated Clinical Deficits J Pediatr 2011 10.1016/j.jpeds.2011.04.008 21621223

[B2] Korf BR Malignancy in neurofibromatosis type 1 Oncologist 2000 5 6 477 485 10.1634/theoncologist.5-6-477 11110599

[B3] Gutmann DH Aylsworth A Carey JC Korf B Marks J Pyeritz RE Rubenstein A Viskochil D The diagnostic evaluation and multidisciplinary management of neurofibromatosis 1 and neurofibromatosis 2 JAMA 1997 278 1 51 57 10.1001/jama.278.1.51 9207339

[B4] Fois A Calistri L Balestri P Vivarelli R Bartalini G Mancini L Berardi A Vanni M Relationship between cafe-au-lait spots as the only symptom and peripheral neurofibromatosis (NF1): a follow-up study Eur J Pediatr 1993 152 6 500 504 10.1007/BF01955059 8335018

[B5] Akcali Y Ceyran H Hasdiraz L Chest wall deformities Acta Chir Hung 1999 38 1 1 3 10439083

[B6] Le LQ Parada LF Tumor microenvironment and neurofibromatosis type I: connecting the GAPs Oncogene 2007 26 32 4609 4616 10.1038/sj.onc.1210261 17297459 PMC2760340

[B7] Friedrich RE Schmelzle R Hartmann M Mautner VF Subtotal and total resection of superficial plexiform neurofibromas of face and neck: four case reports J Craniomaxillofac Surg 2005 33 1 55 60 10.1016/j.jcms.2004.08.004 15694151

[B8] Needle MN Cnaan A Dattilo J Chatten J Phillips PC Shochat S Sutton LN Vaughan SN Zackai EH Zhao H Prognostic signs in the surgical management of plexiform neurofibroma: the Children's Hospital of Philadelphia experience, 1974-1994 J Pediatr 1997 131 5 678 682 10.1016/S0022-3476(97)70092-1 9403645

[B9] Friedrich RE Schmelzle R Hartmann M Mautner VF Subtotal and total resection of superficial plexiform neurofibromas of face and neck: four case reports J Craniomaxillofac Surg 2005 33 1 55 60 10.1016/j.jcms.2004.08.004 15694151

[B10] Ravitch MM The Operative Treatment of Pectus Excavatum Ann Surg 1949 129 4 429 444 10.1097/00000658-194904000-00002 17859324 PMC1514034

[B11] Kelly RJ Pectus excavatum: historical background, clinical picture, preoperative evaluation and criteria for operation Semin Pediatr Surg 2008 17 3 181 193 10.1053/j.sempedsurg.2008.03.002 18582824

[B12] Schaarschmidt K Kolberg-Schwerdt A Dimitrov G Straubeta J Submuscular bar, multiple pericostal bar fixation, bilateral thoracoscopy: A modified Nuss repair in adolescents J Pediatr Surg 2002 37 9 1276 1280 10.1053/jpsu.2002.34982 12194116

[B13] Nuss D Minimally invasive surgical repair of pectus excavatum Semin Pediatr Surg 2008 17 3 209 217 10.1053/j.sempedsurg.2008.03.003 18582827

[B14] Liu WY Xu B Ji Y Wang YX Qin DR [Non-thoracoscopic Nuss procedure for correction operation of pectus excavatum ] Zhonghua Wai Ke Za Zhi 2008 46 8 567 569 18844047

[B15] Ji Y Liu W Xu B Qin D [Non-thoracoscopic minimally invasive Nuss procedure for correction of recurrent pectus excavatum] Zhongguo Xiu Fu Chong Jian Wai Ke Za Zhi 2008 22 10 1213 1217 18979881

[B16] Okamura T Nagase Y Mitsui F Shibairi M Utsumi K Watanabe H Simultaneous repair of pectus excavatum and congenital heart defect in adults by using the convex bar Ann Thorac Surg 2004 77 5 1827 1829 10.1016/S0003-4975(03)01244-X 15111201

[B17] Metzelder ML Ure BM Leonhardt J Grigull L Khelif K Petersen C Impact of concomitant thoracic interventions on feasibility of Nuss procedure J Pediatr Surg 2007 42 11 1853 1859 10.1016/j.jpedsurg.2007.07.011 18022435

